# Optical Monitoring of the Production Quality of Si-Nanoribbon Chips Intended for the Detection of ASD-Associated Oligonucleotides

**DOI:** 10.3390/mi12020147

**Published:** 2021-02-03

**Authors:** Kristina A. Malsagova, Tatyana O. Pleshakova, Vladimir P. Popov, Igor N. Kupriyanov, Rafael A. Galiullin, Andrey F. Kozlov, Ivan D. Shumov, Anna L. Kaysheva, Fedor V. Tikhonenko, Alexander I. Archakov, Yuri D. Ivanov

**Affiliations:** 1 Laboratory of Nanobiotechnology, Institute of Biomedical Chemistry, 119121 Moscow, Russia; t.pleshakova1@gmail.com (T.O.P.); rafael.anvarovich@gmail.com (R.A.G.); afkozlow@mail.ru (A.F.K.); shum230988@mail.ru (I.D.S.); kaysheva1@gmail.com (A.L.K.); alexander.archakov@ibmc.msk.ru (A.I.A.); yurii.ivanov.nata@gmail.com (Y.D.I.); 2 Rzhanov Institute of Semiconductor Physics, Siberian Branch of Russian Academy of Sciences, Laboratory of Silicon Material Science, 630090 Novosibirsk, Russia; popov@isp.nsc.ru (V.P.P.); ftikhonenko@gmail.com (F.V.T.); 3 Sobolev Institute of Geology and Mineralogy, Siberian Branch of Russian Academy of Sciences, Laboratory of Experimental Mineralogy and Crystallogenesis, 630090 Novosibirsk, Russia; spectra@igm.nsc.ru

**Keywords:** autism spectrum disorders, microRNA, silicon nanoribbon, sensor chip, DNA oligonucleotides, Raman spectroscopy

## Abstract

Gas-phase etching and optical lithography were employed for the fabrication of a silicon nanoribbon chip (Si-NR chip). The quality of the so-fabricated silicon nanoribbons (Si-NRs) was monitored by optical Raman scattering spectroscopy. It was demonstrated that the structures of the Si-NRs were virtually defect-free, meaning they could be used for highly sensitive detection of biological macromolecules. The Si-NR chips were then used for the highly sensitive nanoelectronics detection of DNA oligonucleotides (oDNAs), which represent synthetic analogs of 106a-5p microRNA (miR-106a-5p), associated with the development of autism spectrum disorders in children. The specificity of the analysis was attained by the sensitization of the Si-NR chip sur-face by covalent immobilization of oDNA probes, whose nucleotide sequence was complementary to the known sequence of miR-106a-5p. The use of the Si-NR chip was demonstrated to al-low for the rapid label-free real-time detection of oDNA at ultra-low (~10^−17^ M) concentrations.

## 1. Introduction

Autism spectrum disorders (ASDs) are general developmental disorders characterized by persistent deficits in the ability to maintain and initiate social interactions and connections and by limited interests and frequently repetitive behaviors. ASDs develop in childhood and continue into adolescence and adulthood. In most cases, these disorders manifest themselves in the first five years of life. ASDs are often accompanied by other disorders, including epilepsy, depression, anxiety, and attention deficit hyperactivity disorder [1,2,3]. The intelligence level of those experiencing ASDs varies from severe cognitive deficits to high cognitive abilities. According to the estimation by Elsabbagh et al., world-wide, one child in every 160 suffers from an ASD [4]. ASDs appear due to brain disorders and lead to changes at the metabolomic, transcriptomic, and proteomic levels.

At present, a number of research groups are attempting to use mass spectrometry-based (MS) analysis for the revelation of changes in metabolomic and proteomic profiles of patients with ASDs [5,6,7,8,9,10]. It should be emphasized that, to date, the concentration limit of protein detection in plasma that is attainable by MS is not high, at between 10^−12^ M and 10^−9^ M [11]. Such sensitivity is obviously insufficient for the detection of low- and ultra-low-abundant proteins and nucleic acids at early stages of ASD development.

The revelation of ASDs is hindered by the absence of a medical test (based, for in-stance, on blood screening), which would enable the diagnosis of these disorders. In order to diagnose ASDs currently, doctors evaluate the developmental history and behavior of the child. In some cases, ASDs can be determined in children that are 18-months-old and younger. By the age of two, a diagnosis made by an experienced specialist can be considered to be quite reliable [12]. In many cases, however, the final diagnosis is unknown until the patient becomes much older. This means that many children with ASDs do not receive the necessary medical aid in time. Late revelation of ASDs in children leads to the development of severe forms of these diseases. This is why the development of novel diagnostic approaches, which allow for the early diagnosis of ASDs in children, is an urgent task for modern biomedical research.

One of the directions in modern biomedical research is the development of highly sensitive methods for the early revelation of biomolecular markers of diseases [13]. In particular, nanowire-based detection allows one to reveal proteins and nucleic acids in bio-logical fluids at low and ultra-low (<10^−14^ M) concentrations, corresponding to early stages of disease development [13,14,15]. Besides high sensitivity, nanowire-based systems allow one to perform label-free analysis in real time. The high sensitivity of nanowire-based detectors is determined by the high surface-to-volume ratio of nanowire sensor elements [16]. One of the directions in the development of such biosensors is, accordingly, the fabrication of silicon nanoribbon (Si-NR) chips with the smallest dimensions of NRs. The theoretical detection limit, attainable with a nanoribbon (NR) biosensor, can be as low as single molecules per sensor element [17]. At the same time, the NR sensor elements must be free of any defects to reach such low detection limits. Thus, the problem is the fabrication of defect-free NR sensors, which requires proper quality control of the fabricated NRs. Optical nondestructive spectroscopy represents the most convenient method for monitoring the quality of materials used for the fabrication of NRs. To date, measurements of Raman light scattering on silicon nanostructures have been reported in a number of papers [18,19,20]. Herein, we employed Raman spectroscopy in the process of fabricating silicon chips based on silicon-on-insulator (SOI) structures intended for the highly sensitive detection of biological macromolecules. The SOI-based Si-NR chips were then used for the detection of a synthetic oDNA analog of ASD-associated microRNA.

The detection of microRNA is relevant due to recent studies considering the use of microRNAs as promising biomolecular markers associated with the development of various pathologies in humans [21,22,23]. In this way, the use of miR1246, miR-4634, miR1307-3p, miR-6875-5p, miR-6861-5p, and miR-10b as potential markers of breast cancer has been discussed [23,24]. With regard to ASDs, in the literature, a number of microRNAs, including miR-106a, miR-494, miR-19a, miR-19b, etc. [25], were reported to be associated with these disorders. A twofold increase in the miR-106-5p serum level in children suffering from ASD—as compared with that in the control group—was reported [26]. It should be emphasized that the structure of microRNAs is much more stable in comparison with that of proteins. Owing to the high sensitivity of the Si-NR, early detection of ASD-specific miRNA biomarkers is possible even when their concentration in serum is low.

In the present work, Si-NR chips were fabricated on the basis of SOI structures. The optical spectroscopy method was employed for defect detection in the SOI structures. The fabricated defect-free Si-NR chips were subsequently employed for the biospecific detection of synthetic oDNA analogs of miR-106a-5p microRNA, associated with the development of ASDs in children [26]. The surface of the Si-NR chips was sensitized by covalent immobilization of oDNA probes, whose oligonucleotide sequence was complementary to that of the target oDNAs. It has been experimentally demonstrated that the nanowire-based detection with oDNA-sensitized Si-NR chips can be successfully employed for the detection of complementary oDNAs (representing synthetic analogs of microRNAs) in buffer solution, attaining a 3.3 × 10^−17^ M concentration detection limit.

Thus, the approach proposed herein will allow for future serological diagnosis of ASDs by performing the revelation of circulating non-coding microRNAs in biological samples. Further development of this approach, which is based on the use of oDNA-sensitized Si-NR chips, can allow for the elaboration of an adequate diagnostic system for the early diagnosis of ASDs on the basis of quantitative data obtained in the course of serological analysis. This approach primarily utilizes direct label-free, amplification-free detection of target biomarker molecules with high concentration sensitivity in real time. These key features will allow one to correctly and objectively perform timely diagnoses of ASDs in children.

## 2. Materials and Methods

### 2.1. Chemicals

Potassium phosphate monobasic (KH_2_PO_4_) and (3-aminopropyl) triethoxysilane (APTES) were purchased from Sigma-Aldrich (St. Louis, MO, USA). Glycine, toluene, and hydrofluoric acid (HF) were purchased from Reakhim (Moscow, Russia). Potassium tetraborate was purchased from Acros Organics (Geel, Belgium). Isopropanol was purchased from Acros Organics (Geel, Belgium). 3,3′-dithiobis (sulfosuccinimidyl propionate) (DTSSP) was purchased from Pierce (Waltham, MA, USA). Ultrapure deionized water was obtained with a Simplicity UV Water Purification System (Millipore, Molsheim, France).

### 2.2. Oligonucleotides

The oDNA probes, employed in our experiments, were purchased from Evrogen (Moscow, Russia). The nucleotide sequences of these oDNAs are listed in Table 1.

### 2.3. Fabrication of Si-NR Chips

The layout and characteristics of Si-NR chips, employed herein, were described in detail in our previous papers [27,28,29].

**Figure 1:**
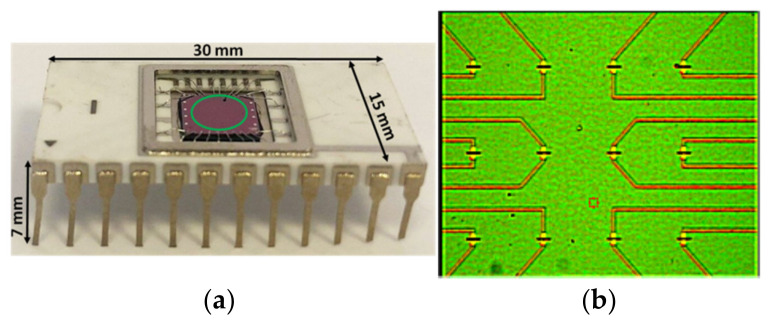
Photographic images of (**a**) a mounted silicon nanoribbon (Si-NR) chip (top view; green circle indicates the sensitive area of the chip), and (**b**) sensitive area of the chip, containing an array of twelve NRs (**b**).

“Silicon-on-insulator” (SOI) wafers were fabricated using a technology similar to Smart Cut. This technology was based on the hydrogen-induced transfer of silicon layers onto a handle wafer. The differences from Smart Cut technology consisted of the following: buried oxide (BOX) was not subjected to hydrogen implantation—as in the Smart Cut process—and the interface between the BOX and the top silicon layer was a bonded inter-face. This approach to SOI formation allows one to obtain Si/SiO_2_ systems with low defect levels and, thus, provides parameter stability of the devices fabricated on the basis of these systems. The method of the SOI structure formation has been described previously [30]. The concentration of free charge carriers in the top Si layer of the SOI wafers was (2 to 6) × 10^16^ cm^−3^. The total initial thickness of the SOI layers was 500 nm, with the buried-oxide thickness being 300 nm. The thickness of the top Si layer was then reduced down to 32–24 nm by repeatedly performed thermal oxidation/oxide removal steps. Treatment of the samples in hydrofluoric acid was then used to remove the oxide layer. Afterward, the obtained SOI samples with nanometer-thick top Si layers were patterned by optical lithography in order to obtain mesa-structures shaped as 3 μm wide Si strips of 10 μm length. Within the contact pad area, a 250 nm thick poly-Si layer was deposited onto the SOI layer using low pressure chemical vapor deposition (LPCVD). Phosphorus (or boron) ions were then implanted into the deposited poly-Si layer, and the samples were given either a 20 min anneal at 950 °C or a 20 s anneal at 1075 °C to activate the implants. The use of poly-Si layers, which extended over the con-tact pads, allowed us to simultaneously prepare structures with top lateral poly-Si gates (TG). Similar to the buried oxide (BOX) thickness, the gate oxide thickness was 300 nm. Metallization with aluminum was then applied and, subsequently, electron lithography and XeF_2_ gas etching were used to reduce the NR width in the central part of the structure to 200 ± 30 nm. The Si/SiO_2_ etching selectivity was 1200 to 1500. Also, plasma-enhanced chemical etching in SF_6_:CFCl_3_ gas mixture was used. In the latter case, the bias potential of the substrate with respect to the plasma during the etching was within 10 V. This allowed us to organize the conditions close to those of the gas etching [31] and to minimize the radiation damages to SOI induced by ion bombardment. Finally, a post-metallization annealing in forming gas at 420 °C was carried out (Figure 1).

The sensor elements of the Si-NR chips (nanoribbons, NRs) were fabricated by nanostructuring on SOI wafers with n-type conductance. The Si-NR chips had the following dimensions: length *l* = 30 mm, width *w* = 15 mm, and height *h* = 7 mm (Figure 1a). The thickness of the cutoff layer was 32 nm, while the BOX thickness was 300 nm. Since NR fabrication is a low-temperature process, it does not cause degradation of the SOI structure parameters. The formation of NRs has been described in detail elsewhere [30]. The width and length of each NR were 3 µm and 10 µm, respectively, while their number on a single Si-NR chip was 12 (Figure 1b). Accordingly, the ratio of the working area of a single 3 µm wide NR to the total area of the Si-NW chip was 0.5 × 10^−5^.

Optical control of the quality of Si-NR chips was performed by Raman spectroscopy with a Horiba Jobin-Yvon LabRam HR800 spectrometer (HORIBA, Kyoto, Japan). A built-in Olympus BX41 microscope (Olympus Corp., Tokyo, Japan; objective focal distance 1 mm; N.A. = 0.65; analyzed area diameter< 3 µm) was used for the analysis of submicron areas of the channels of field-effect transistors (FETs). A confocal optical scheme, realized using this microscope, allowed us to achieve maximum detail. The latter was necessary as the dimensions of the NR sensor elements were very small. At the same time, this optical scheme allowed us to maintain a high speed of image acquisition upon excitation with a ~1.0 mW argon laser beam. This allowed us to prevent heating of the coated sensor surface.

### 2.4. SEM Analysis

Scanning electron microscopy (SEM) represents an efficient and informative method of surface structure analysis. In our study, this nondestructive method allowed us to rap-idly perform quantitative morphological analysis of the NR sensor surface. The use of a field emission source for the formation of a diagnostic beam with energies of 10 and 20 keV allowed for the visualization of relief inhomogeneities within one monolayer for the analysis of the chemical composition of the surface during the development of gas-phase etching modes and for the determination of the electrical insulation of nanolayers and the presence of defects in the buried oxide (BOX) using the induced current technique. SEM measurements of linear dimensions of the microrelief of the NR sensor surface with a resolution down to 1 nm were performed with a LEO-1430 scanning electron microscope equipped with an attachment for energy dispersive analysis (EDX; Zeiss, Germany).

**Figure 2:**
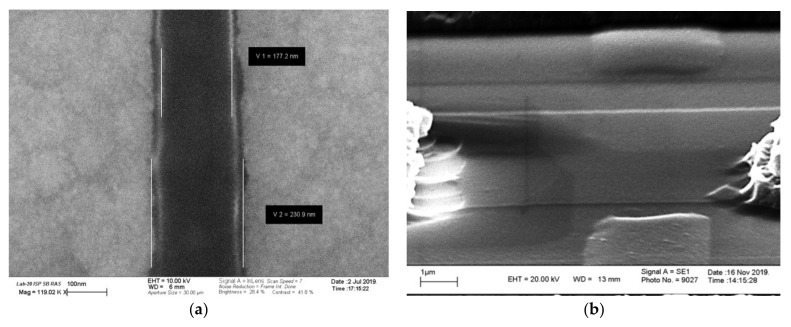
Typical scanning electron microscopy (SEM) images of NRs with a width of 200 nm ((**a**), top view) and 3µm ((**b**), at a 60° angle to the surface).

A set of Si-NR chips bearing 10 µm long NRs of 200 nm to 6 µm width and 24 nm to 32 nm thickness were developed. Figure 2a displays typical SEM images of NRs of 200 nm and 3 µm width. Figure 2b displays the image of a 3 µm wide NR showing defects in the source and drain areas and polysilicon lateral gate electrodes.

The SEM images in Figure 2 indicate the absence of significant structural defects in the NRs fabricated in both the silicon layer and the insulating buried oxide layer (BOX).

### 2.5. Functionalization of Si-NR Chip Surface

**Figure 3:**
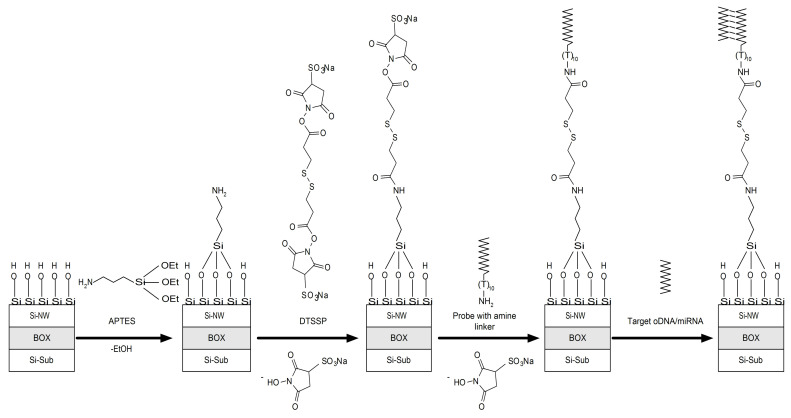
Schematic representation of Si-NR surface functionalization and target molecule detection.

Functionalization of the Si-NR chip surface provides biospecificity of the detection of target molecules and stability of the signal received from the NR sensor elements. NR sur-face functionalization is a two-step process comprised of chemical modification and sensitization with molecular probes (Figure 3).

Chemical modification included a preliminary treatment of the Si-NR chip surface and its silanization with APTES. During the preliminary treatment, the Si-NR chip was cleaned of mechanical and organic contaminants by rinsing with isopropanol. This was followed by treatment of the Si-NR chip surface with a solution containing HF and ethanol to remove the native oxide layer formed during the chip storage. After that, the Si-NR chip was treated in a UV ozone cleaner ProCleaner™ Plus ozonator (Ossila Ltd., Sheffield, UK) for 1 h in order to form hydroxyl groups on its surface. The so-treated Si-NR chip was then silanized in APTES (0.1%) vapors for 20 h at room temperature in order to form a layer with terminal amine groups on its surface. Toluene was used as a solvent. The amine groups were necessary for the covalent immobilization of molecular probes onto the chip surface. Finally, the Si-NR chip surface was treated with ethanol and dried. To provide biospecificity of the detection of target oDNAs, the Si-NR chip surface was sensitized by covalent immobilization of molecular probes (Table 1). To immobilize the oDNA probes onto the Si-NR chip surface, the latter was activated with DTSSP crosslinker. For this purpose, 1 µg of DTSSP was dissolved in a mixture containing ethanol (6 µL) and 50 mM potassium phosphate buffer (14 µL). This solution was incubated in a shaker at 10 °C and 600 rpm for 10 min. The obtained crosslinker solution was then immediately used for Si-NR surface activation. After the activation, 3 nL microdrops of 1 μM solutions of oDNA probes in 50 mM KP buffer (pH 7.4) were precisely dispensed onto the surface of each single NR to be sensitized with a Piezorray low volume high accuracy dispensing system (PerkinElmer, Inc., Waltham, MA, USA). The immobilization time of the oDNA probes was 20 min. In our experiments, several NRs on the sensor chip were sensitized with oDNA probes whose oligonucleotide sequence was complementary to that of the target oDNAs (Table 1, row 1); these NRs were used as working sensors. Other NRs were sensitized with oDNAs, specifically against breast cancer-associated hsa-miR-4634, and these NRs were used as control sensors (Table 1, row 2). The solutions of oDNAs to be immobilized were incubated on the chip surface for 0.5 h at 15 °C and 80% relative air humidity. After that, the Si-NR chip was rinsed with ultrapure water.

### 2.6. Preparation of Buffered Solutions of Target oDNAs

Solutions of target oDNAs with a concentration of 10^−16^ M were prepared from the initial 100 µM solution in 50 mM KP buffer (pH 7.4) by sequential tenfold dilution with working buffer (1 mM KP, pH 7.4). On each dilution step, the oDNA solution was incubated in a shaker for 0.5 h at 10 °C and 600 rpm. The so-prepared target oDNA solution was used for biosensor measurements immediately after preparation.

### 2.7. Electrical Measurements

Electrical measurements were performed with a Keithley 6487 picoampermeter (Keithley, Solon, OH, USA). In the measurements, the substrate of the SOI structures was used as a transistor gate. The dependence of the drain-source current on gate voltage *I*_ds_ *(V*_g_*)* for n-type SOI NRs was obtained at gate voltage *V*_g_ from 0 to 40 V and drain-source voltage *V*_ds_ = 0.2 V.

Time dependencies of the current *I*_ds_(*t*) were recorded in real time at *V*_g_ = 30 V and *V*_ds_ = 0.2 V. The results obtained were presented in the form of *I*_ds_(*t*) curves, representing the differential signal between the working NR (with immobilized probe_1) and control NR (with immobilized probe_2). A detailed schematic of electrical signal processing has been presented in [27].

The biosensor measurements were performed in the following way. The sample solution to be analyzed (150 µL in 1 mM KP buffer), containing 10^−16^ M of the target oDNA, was added into a measuring cell containing 300 µL of pure buffer. That is, the resulting concentration of the target oDNA was decreased by three times. Control experiments were performed in the same way, but using pure oDNA-free buffer as the sample solution.

To avoid problems associated by the Debye screening effect (*λ*_D_) [32,33], the oDNA detection was performed in a low-salt (1 mM) buffer.

## 3. Results

### 3.1. Optical Control of Si-NR Chip Quality

**Figure 4:**
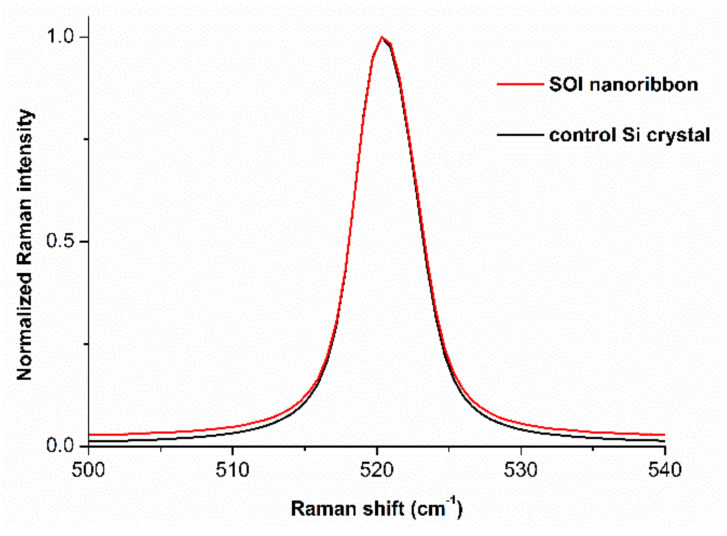
Typical normalized Raman spectra, obtained for the control silicon crystal (Si) and for the silicon nanoribbon (SOI). The spectra were excited with a 514 nm Ar^+^ laser beam.

Monitoring of the quality of SOI structures of Si-NR chips was performed by optical Raman spectroscopy. The spectra were excited with a 514 nm Ar^+^ laser beam. Figure 4 displays typical normalized Raman spectra, obtained for a silicon crystal (Si) and for a silicon nanowire (SOI). Upon the spectrum acquisition, the laser beam was directed along the <100> axis of the SOI structure forming silicon. The pure silicon crystal was used as a control structure. Accordingly, the characteristics of the silicon nanowire were compared to those of the control silicon crystal.

**Figure 5:**
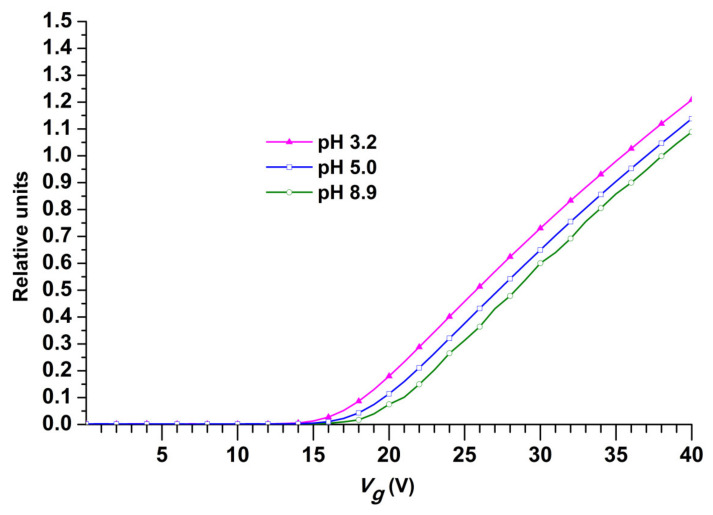
Typical current–voltage characteristics, obtained for the Si-NR chip with n-type conductance in media with various pH: 1 mM glycine buffer (pH 3.2, purple line and triangles), 1 mM borate buffer (pH 8.9, green line and white circles), and ultrapure water (pH 5.0, blue line and white squares). Experimental conditions: *V*_g_ from 0 to 40 V; *V*_ds_ = 0.2 V; 300 μL volume of liquid in the cell.

As seen from Figure 4, the normalized spectra curves, obtained for both the SOI nano-ribbon structure and the control silicon crystal, have virtually the same shape. Thus, one can characterize the chip structure as free of stresses and defects, such as inhomogeneities in the surface relief or holes in the silicon layer or in the BOX layer, causing current leak-age or absence of field-induced modulation of the NR conductance in the crystal structure. Such defects induce tensile and compressive stresses in the SOI structure, and the shape of the Raman spectrum curve changes to an inhomogeneously broadened peak instead of a single Lorentzian line. The approximation of such a curve is possible by a combination of several curves, which is verified by comparison with a bulk control crystal. In our case, the coincidence in the shape of Raman spectra obtained for the control silicon crystal (Si) and for the silicon nanowire (SOI), shown in Figure 5, indicates the absence of any considerable stresses and defects in the structure of the nanowires formed on the Si-NR chips. Accordingly, the Si-NR chips can be employed in highly sensitive biosensor experiments.

### 3.2. Tests of the Si-NR Chip Performance

The drain-source current (*I*_ds_) through the sensor structures of a Si-NR chip is known to be dependent on the pH of the analyzed medium [34,35,36]. Such a dependence takes place owing to a modulation of the drain-source current upon the adsorption of ions from the analyzed solution onto the surface of the sensor structures. Therefore, it is important to determine whether the performance of the Si-NR chip with APTES-modified surface is ac-curate at various pH levels of the analyzed solution. Figure 5 displays typical current–voltage characteristics obtained for the Si-NR chip with n-type conductance in media with various pH levels. Namely, 1 mM glycine buffer (pH 3.2), ultrapure water (pH 5.0), and 1 mM borate buffer (pH 8.9) were used in this test.

The curves shown in Figure 5 clearly indicate a shift of the NR characteristic curves to the right (the green curve relative to the blue curve) upon increasing the pH from 5.0 to 8.9. On the contrary, decreasing the pH from 5.0 to 3.2 induces a shift of the characteristic curves to the left (the purple curve relative to the blue curve in Figure 5). The increase in the n-type NR’s conductance with decreasing pH of the medium correlates with the data on the increase in the zeta potential of APTES-modified SiO_2_ surface reported by Knopfmacher et al. [34]. This indicates the correct operation of the Si-NR chip.

### 3.3. Biospecific Detection of oDNAs in Buffer with the Si-NR Chip

An oDNA-sensitized Si-NR chip was employed for the highly sensitive detection of the complementary target oDNA in buffer at ultra-low (subfemtomolar) concentration. Since Si-NRs in the structure of an n-type field-effect transistor were used, the hybridization of target oDNAs with NR-immobilized probe oDNAs was preceded by the adsorption of negatively charged oDNA molecules onto the NR surface, leading to an accumulation of an excessive negative charge on this surface (which represented a virtual gate). The ad-sorption of negative charge should have led to a decrease in the NR conductance at constant gate voltage *V*_g_, and this was what we observed in our experiments.

**Figure 6:**
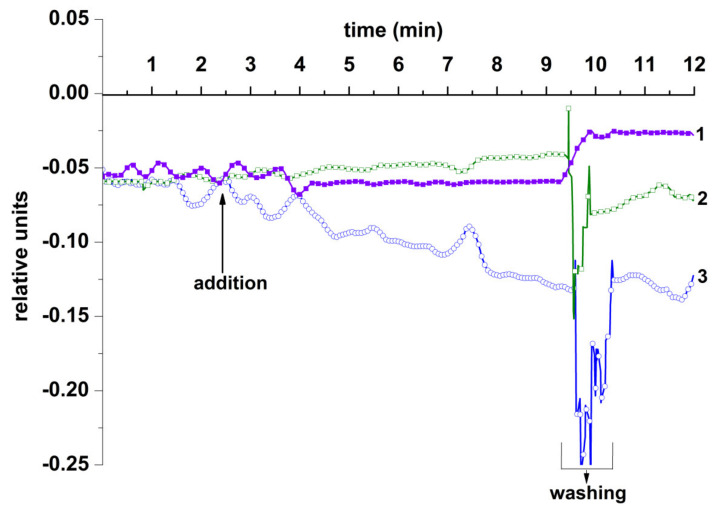
Typical time dependencies of the biosensor signal, obtained upon the detection of synthetic oDNA analog of spectrum disorder (ASD)-associated miRNA in 1 mM KP buffer solution with an n-type Si-NR chip: control experiment with the use of pure oDNA-free 1 mM KP buffer (curve 1); the signal from the NR, sensitized with oDNA probe_2 (specific against breast cancer markers) upon the addition of ASD-associated oDNA (curve 2); the signal from the NR, sensitized with oDNA probe_1 (specific against ASD) upon the addition of ASD-associated oDNA (curve 3). Experimental conditions: concentration of the target oDNA solution in the sample 10^−16^ M; final concentration of the target oDNA in the measuring cell 3.3 × 10^−17^ M. *V*_g_ = 30 V; *V*_ds_ = 0.2 V; 150 μL volume of added sample; 450 μL total solution volume in the cell.

Figure 6 displays the typical time dependence of the biosensor signal, recorded in the analysis of 3.3 × 10^−17^ M ASD-associated oDNA solution in KP buffer (pH 7.4).

The curve shown in Figure 6 indicates a decrease in the conductance of the oDNA-sensitized Si-NR chip upon addition of the solution of target oDNA. This decrease in the n-type chip conductance occurs due to the formation of complexes of negatively charged target oDNA molecules with chip-immobilized molecular probes. In addition, it should be emphasized that no considerable change in the biosensor signal level was observed after the target oDNA solution was replaced with pure KP buffer. This indicates a very slow dissociation of the probe/target complexes formed on the chip surface. The specificity of the detection with the oDNA-sensitized Si-NR chip was also studied. For this purpose, the following parameters were tested: (1) the sensitivity of the Si-NR chip to the addition of pure oDNA-free 1 mM KP buffer (Figure 6, curve 1), and (2) the signal from the NR sensitized with breast cancer-associated oDNA (probe_2) upon the addition of ASD-associated oDNA (Figure 6, curve 2). As seen from the curves shown in Figure 6, no significant changes in the NR conductance are observed in these tests. This indicates that the Si-NR chip allows for the specific detection of oDNA molecules.

## 4. Discussion

Our present research is aimed at the fabrication of Si-NR chips with subsequent control of their quality in order to determine whether they can be used for the highly sensitive detection of ASD-associated nucleic acid biomarkers at subfemtomolar (<10^−15^ M) concentrations.

In the “top-down” technology employed in our present study, stresses, as well as de-formations and other defects, can appear in the fabricated nanowire structures. The presence of such defects in the structure of NR sensors causes unpredictable changes in their parameters and impedes the sensitivity attainable with these sensors since they are char-bacterized by a very high surface-to-volume ratio [16]. That is, the parameters of the sensors, required for their application in the early revelation of biomarkers, become unattainable if the sensor structures are defective. It should be reemphasized that early diagnosis of diseases requires the use of highly sensitive detection systems that are able to overcome the 1 fM (10^−15^ M) concentration sensitivity threshold [13]. This is why a proper control of the quality of the fabricated sensor structures is strongly required. In this regard, optical Raman spectroscopy is a very convenient method that allows for rapid nondestructive testing of the fabricated NR sensor structures.

In the present work, Si-NR chips were fabricated using a CMOS-compatible technology by gas-phase reduction and lithography. The absence of stresses and defects in the formed SOI structures were confirmed by optical Raman spectroscopy. Namely, Raman spectra were obtained for the NR of the SOI structure. These spectra were then compared with those obtained for the control silicon crystal. The spectra were excited by Ar^+^ laser beam at 514 nm wavelength and directed along the normalized spectra to the surface of the investigated structures.

MicroRNAs represent non-coding RNAs comprised of 21 to 25 nucleotides. These RNAs are currently considered as potential epigenetic diagnostic markers. Since the sequence of the majority of microRNAs is conserved [37], the use of microRNAs as diagnose-tic and prognostic markers is promising. Herein, a synthetic DNA analog (oDNA) of ASD-associated microRNA miR-106a-5p [26] was used as an object for testing the performance of the Si-NR chips under the conditions of highly sensitive biosensor-based detection ap-plications. The surface of the Si-NR chips was sensitized with covalently immobilized oDNA molecular probes to provide biospecific detection of the target oDNAs. The results of our experiments have indicated that the use of such chips with defect-free NR structures allows one to perform highly sensitive label-free detection of target oDNA molecules in real time at ultra-low (3.3 × 10^−17^ M) concentration. To date, the possible cause of such a high sensitivity of Si-NR chip-based biosensors remains an open question. To answer this question, a laborious calculation of electrohydrodynamics [38] of the delivery of analyte molecules to the chip surface is required. In the literature, surface potential has been shown to be the key factor determining protein binding with the surface [39].

## 5. Conclusions

Silicon nanoribbon (Si-NR) chips were fabricated using CMOS-compatible “top-down” technology. By using optical Raman spectroscopy, the nanowire sensor structures were confirmed to be virtually defect-free. Using the example of synthetic DNA analogs of ASD-associated microRNA miR-106a-5p, it was experimentally demonstrated that these Si-NR chips allowed for highly sensitive label-free, real-time detection of target molecules. The oDNA detection limit attained was as low as 3.3 × 10^−17^ M. The results obtained indicate that nanowire-based detection is a promising tool for diagnostic applications aimed at the early revelation of ASDs in children.

## Figures and Tables

**Table 1:** Complements of oDNA and miRNA.

No.	oDNA Probe	oDNA Sequence Complementary to the Probe («cs», Complementary Sequence)	Corresponding miRNA
1.	(NH_2_)-(T)_10_-CTACCTGCACTGTAAGCACTTTT (probe_1)	AAAAGTGCTTACAGTGCAGGTAG	miR-106a-5p [25]
2.	(NH_2_)-(T)_10_-CGGCGCGACCGGCCCGGGG (probe_2) (control)	CCCCGGGCCGGTCGCGCCG	hsa-miR-4634 [23]

## Data Availability

The datasets generated during and/or analyzed during the current study are available from the corresponding author on reasonable request.
